# Acute Gastrointestinal Bleeding: A Report of Rare Presentation of Duodenal Metastasis From Cervical Squamous Cell Carcinoma

**DOI:** 10.7759/cureus.16245

**Published:** 2021-07-07

**Authors:** Feroza Patel, Trajan Barrera, Erum Azhar, Syed Atif, Abdul Waheed

**Affiliations:** 1 Family Medicine, WellSpan Good Samaritan Hospital, Lebanon, USA; 2 Public Health Sciences, Penn State University College of Medicine, Hershey, USA; 3 Obstetrics and Gynecology, Maimonides Medical Center, Brooklyn, USA; 4 Family and Community Medicine, Penn State University College of Medicine, Milton S. Hershey Medical Center, Hershey, USA

**Keywords:** cervical cancer screening, pap, hpv infection, radiation and clinical oncology, massive lower gi bleeding

## Abstract

We report a case of a 42-year-old gravida 3, para 4 woman from Puerto Rico with a history of cervical cancer who presented with dizziness, lethargy, and three days of bright red blood per rectum. Imaging evaluation showed a retroperitoneal lymph node mass with secondary metastasis to the duodenum. After she was stabilized with multiple blood transfusions and to mitigate her ongoing bleeding, she was transferred to a tertiary care hospital for possible embolization by interventional radiologists. However, she was deemed a poor candidate for an interventional procedure and decided to ultimately go home on hospice care. This case highlights the rarity of duodenal metastasis presenting as gastrointestinal bleeding due to cervical squamous cell cancer and further reinforces the need for human papillomavirus vaccination and cervical cancer screening. This case study also illustrates that even though cervical cancer rates are low in the United States, it is still deadly in many countries across the globe. As people continue to travel and migrate across borders, the risk of being lost to follow-up is on the rise.

## Introduction

Cancer is the second leading cause of death in the United States [1], with cervical cancer previously being one of the leading causes of death for American women. However, in the last four decades, the number of cases of cervical cancer as well as the death rates from cervical cancer have decreased due to the widespread utilization of pap smears to detect early precancerous changes [2]. Metastatic carcinoma to the duodenum from the uterine cervix through the lymphatic spread and hematogenous spread is extremely rare. Involvement of the lymph nodes at the aortic bifurcation is always accompanied by distant spread [3].

The gastrointestinal (GI) tract has been reported to be involved in approximately 8% of all cervical carcinomas, but in most of those cases, the colon was usually involved through lymphatic spread or local invasion. Small bowel involvement is reported as being exceedingly rare, comprising as little as 0.4% of cervical carcinomas, mostly through para-aortic or mesenteric lymphatic spread [4]. To our knowledge, extra-pelvic spread of the squamous cell cancer of the cervix to the small bowel is only reported in six cases in the literature [5].

## Case presentation

**Figure 1:**
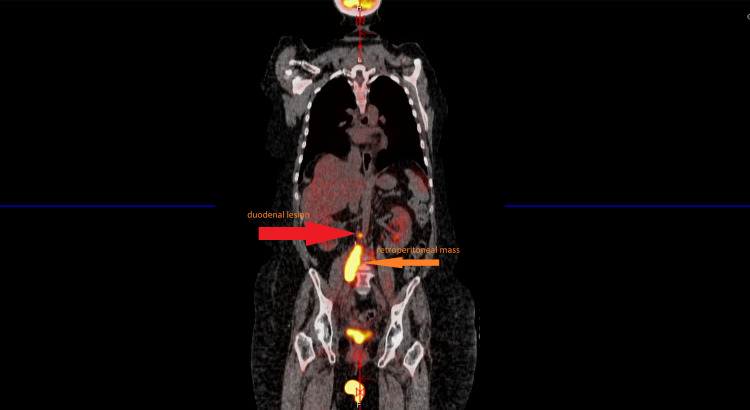
Fludeoxyglucose F-18 positron emission tomography scan showing large hypermetabolic retroperitoneal mass consistent with metastatic cervical cancer, and slightly superior to the large retroperitoneal mass there is a single enlarged mass representing either duodenal spread or lymph node consistent with metastasis

In May 2020, a 42-year-old gravida 3, para 4 female originally from Puerto Rico with a past medical history of systemic lupus erythematosus, expressive aphasia secondary to lupus cerebritis in her 20s, and metastatic squamous cell cervical cancer presented to the Emergency Department with three days of progressive dizziness, light-headedness, and bloody bowel movements with hematochezia. She denied head trauma, falls, vaginal bleeding, hematemesis, or melena, straining, or pain with defecation. A review of her medical chart revealed a distant history of human papillomavirus (HPV) 20 years ago in Puerto Rico and the International Federation of Obstetrics and Gynecology (FIGO) stage 3B squamous cell cervical cancer in 2018. At that time, she was treated with a hysterectomy and bilateral salpingo-oophorectomy, and subsequent radiation and chemotherapy in two different states before relocating to Pennsylvania. Since moving to Pennsylvania in February 2020, she established care locally with Hematology-Oncology and Gynecology-Oncology services. She was found to have confirmed FIGO stage 3B cervical cancer, a retroperitoneal lymph node mass, and secondary spread to the duodenum (Figure 1).

She was scheduled to start outpatient chemotherapy as her prognosis was considered poor by the hematologist/oncologist, and chemotherapy was recommended palliatively.

On physical examination, she was an overweight female in moderate distress with sinus tachycardia, and pelvic exam revealed no vaginal bleeding. Complete blood count showed hemoglobin of 4.9 g/dL (normal range 11.5-15.5 g/dL). Complete metabolic panel revealed mild hyponatremia and prothrombin time/international normalized ratio had a slight elevation. All other labs were within normal limits.

**Figure 2:**
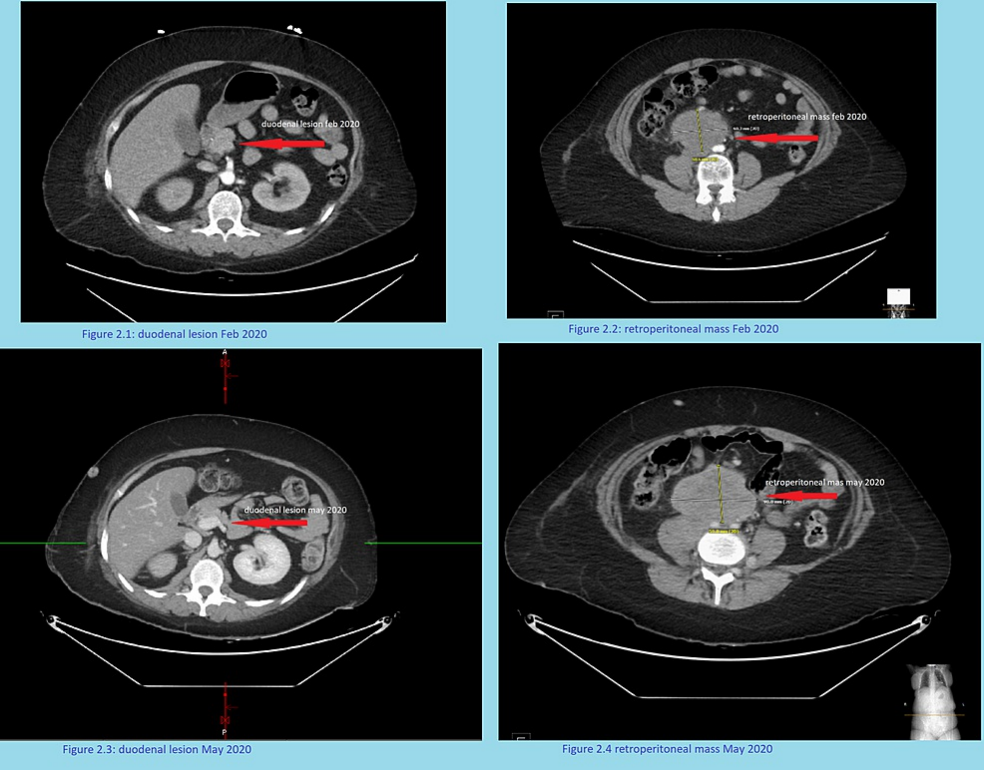
Duodenal and retroperitoneal masses, February 2020-May 2020

Imaging with CT abdomen and pelvis showed a growing retroperitoneal mass that was noted to be larger compared to her earlier imaging studies done in February 2020 (Figure 2).

She was stabilized in the Emergency Department with one unit of packed red blood cells, 2 L of normal saline, and oral pantoprazole. She was urgently admitted to the hospital and given two more units of packed red blood cells on the floor with a hemoglobin transfusion goal of 8-9 g/dL. Intravenous pantoprazole was started, and the on-call gastroenterologist was consulted. Repeat hemoglobin was 7.5 g/dL (normal range 11.5-15.5 g/dL).

**Figure 3:**
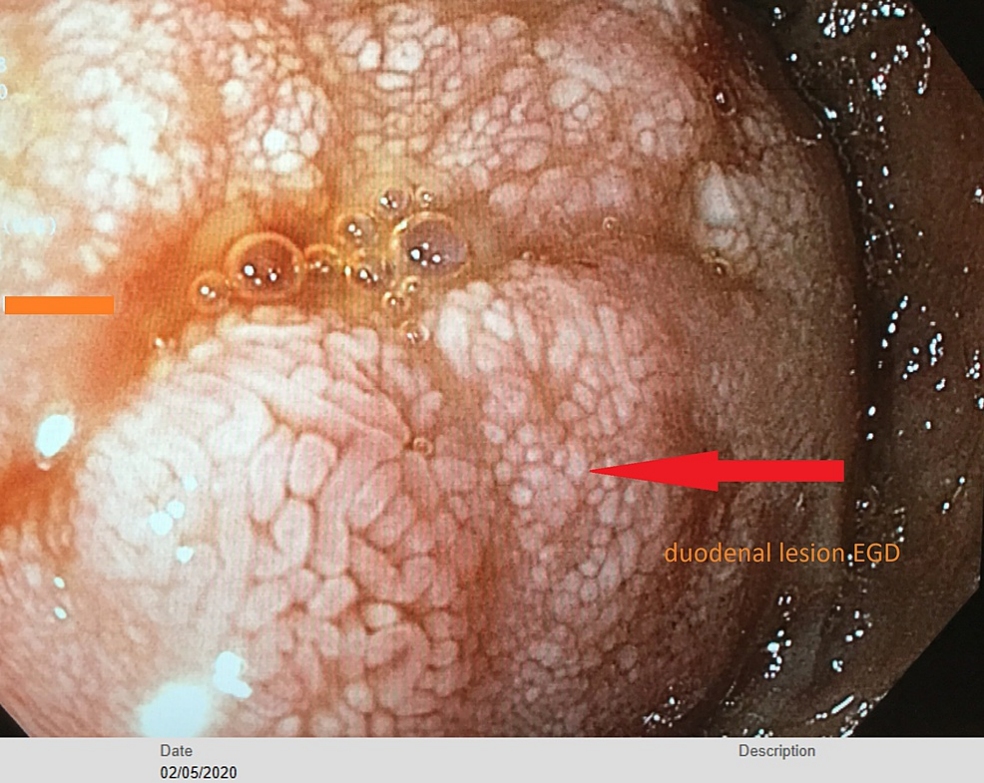
6 cm mass in the second part of duodenum in February 2020 EGD, esophagogastroduodenoscopy

The consulting gastroenterologist noted that she had an upper endoscopy three months ago in February 2020 when she was previously admitted for GI bleeding, which showed a 6 cm bleeding mass in the second part of the duodenum without arteriovenous malformation or ulcers. At that time, the mass was electrocauterized and sprayed with limited benefit and the mass was biopsied and genetic testing revealed it to be likely metastasis from her cervical squamous carcinoma as shown in Figure 3.

During this admission, given the patient's clinical history, the findings were consistent with metastatic cervical squamous carcinoma. Having considered her history, current stability, slow nature of bleed, and poor long-term prognosis, the gastroenterologist decided not to perform an endoscopy at this current visit.

On day 2 of her hospitalization, the patient’s hemoglobin dropped below 7 g/dL and one more unit of packed red blood cells was transfused. Palliative care was consulted given the poor prognosis; however, she changed her code status to do not resuscitate. Interventional radiology (IR) was consulted for possible arterial embolization of the duodenal mass and she was transferred to a tertiary care hospital to accommodate IR embolization services.

**Figure 4:**
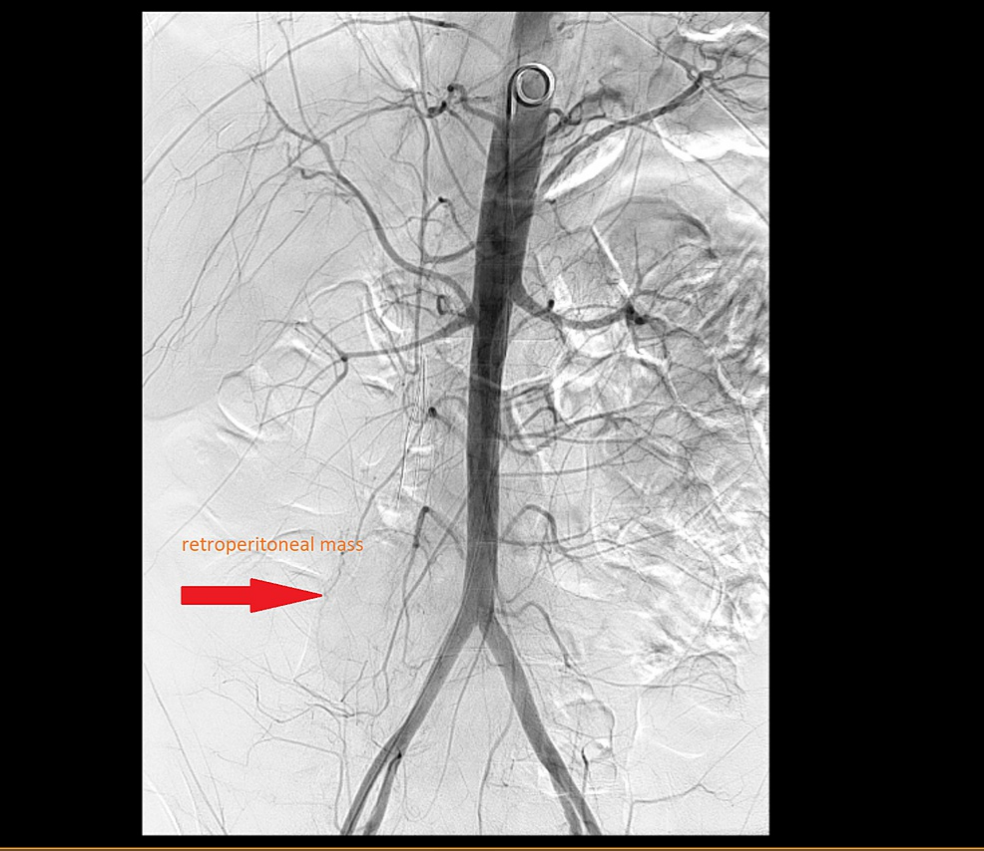
IR arteriogram showing no visible bleeding and obscuring retroperitoneal mass IR, interventional radiology

At the tertiary care hospital, the patient continued to have melena with dropping hemoglobin requiring additional transfusions. Arteriogram failed to identify the source of bleed and imaging was complicated by obstructing retroperitoneal mass as shown in Figure 4. The patient was ultimately deemed a poor surgical candidate for exploratory surgery, and in consultation with the patient and her family, the patient decided on outpatient hospice care.

Ultimately, she was able to go home on hospice to be with her family, with the understanding of the terminal prognosis of her situation so that her family could gradually start the grieving process and be able to say goodbye. She ultimately succumbed to her metastasis within six weeks of going home under the care of her outpatient hospice physician.

## Discussion

Cervical cancer is the leading cause of cancer-related deaths in women in much of Africa. Cervical cancer is also responsible for over 300,000 fatalities worldwide in 2018, with one-third of them in China and India alone [6]. Migrants to the United States prove to be an example of ongoing challenges in vaccination and preventative global medicine [7] as well as problems of transitioning care and the need for increased insurance coverage and access to high-quality healthcare in a new global economy.

Cancer of the cervix characteristically spreads by local and direct invasion of surrounding tissues. Metastasis to a distant site is rare and most commonly occurs via the lymphatic spread, although hematogenous spread may occur in approximately 5% of patients. Though common sites involved in distant extra-pelvic cervical cancer metastasis are the lungs and liver, bowel involvement is usually in the large bowel rather than the small bowel [8]. According to Kanthan et al. who studied metastatic cervical cancer, in 199 patients in a hospital in India, only one case of duodenal spread was found [5].

Patterns of cervical cancer recurrence vary with treatment. After surgery alone, an increase in central pelvic recurrence was seen mostly due to inadequate surgical staging. Distant metastases were seen after radiation alone. On the other hand, after the combined treatment of surgery and radiation, an increase in lateral deep pelvic recurrence was observed mainly due to inadequate radiation doses [9,10]. Lymphatic spread in cervical cancer metastasis generally occurs in an orderly manner, first occurring to the primary lymph nodes followed by the extra-pelvic lymph nodes. The common sites of distant cervical cancer metastasis are the lungs and liver [10,11]. Duodenal metastasis from endocervical carcinoma is quite rare. There is no direct lymphatic connection from the cervix to the small bowel, but rather an indirect route through the para-aortic lymph nodes [6].

## Conclusions

While acute GI bleeding from duodenal metastasis from cervical cancer is indeed rare, practicing physicians should be aware of this uncommon occurrence. This especially rings true in a patient with a history of cervical cancer who presents with sudden-onset lower GI bleed and no other known risk factors. Her case provides a valuable lesson because while cervical cancer metastasis is few and far between in the United States, it is rampant throughout much of the developing world. Given this fact and the fact that surgery and radiation are often ineffective, this case report demonstrates the importance of continued HPV vaccination and routine screening with pap smears for women, especially immigrants who are establishing care in the United States.
